# A Dual-Channel and Frequency-Aware Approach for Lightweight Video Instance Segmentation

**DOI:** 10.3390/s25020459

**Published:** 2025-01-14

**Authors:** Mingzhu Liu, Wei Zhang, Haoran Wei

**Affiliations:** The Higher Educational Key Laboratory for Measuring & Control Technology and Instrumentation of Heilongjiang Province, Harbin University of Science and Technology, Harbin 150080, China; 2220610123@stu.hrbust.edu.cn (W.Z.);

**Keywords:** video understanding, video transformer, visual perception intelligent sensing, video instance segmentation, lightweight

## Abstract

Video instance segmentation, a key technology for intelligent sensing in visual perception, plays a key role in automated surveillance, robotics, and smart cities. These scenarios rely on real-time and efficient target-tracking capabilities for accurate perception and intelligent analysis of dynamic environments. However, traditional video instance segmentation methods face complex models, high computational overheads, and slow segmentation speeds in time-series feature extraction, especially in resource-constrained environments. To address these challenges, a Dual-Channel and Frequency-Aware Approach for Lightweight Video Instance Segmentation (DCFA-LVIS) is proposed in this paper. In feature extraction, a DCEResNet backbone network structure based on a dual-channel feature enhancement mechanism is designed to improve the model’s accuracy by enhancing the feature extraction and representation capabilities. In instance tracking, a dual-frequency perceptual enhancement network structure is constructed, which uses an independent instance query mechanism to capture temporal information and combines with a frequency-aware attention mechanism to capture instance features on different attention layers of high and low frequencies, respectively, to effectively reduce the complexity of the model, decrease the number of parameters, and improve the segmentation efficiency. Experiments show that the model proposed in this paper achieves state-of-the-art segmentation performance with few parameters on the YouTube-VIS dataset, demonstrating its efficiency and practicality. This method significantly enhances the application efficiency and adaptability of visual perception intelligent sensing technology in video data acquisition and processing, providing strong support for its widespread deployment.

## 1. Introduction

Video Instance Segmentation (VIS) [1] represents a novel area of research that builds upon advancements in image-level instance segmentation tasks. Video instance segmentation, a crucial aspect of smart visual sensing, involves identifying and isolating objects in every visual scene and establishing relationships between them to generate a coherent sequence of segmented instance masks. In essence, the process involves assigning identical labels to pixels associated with the same object across successive frames by leveraging analyzed object characteristics within the video [2]. With the wide popularity of smart sensors and surveillance devices, the application scenarios of visually aware smart sensing are expanding, such as automated surveillance, intelligent transport, and environmental monitoring. To address the challenges of real-time data processing in sensor networks, video instance segmentation algorithms not only need to maintain high accuracy in target detection and segmentation but also need to have low computational overhead and efficient processing capability. Nevertheless, issues such as motion artifacts, out-of-focus lenses, partial obstructions, and variations in object appearance frequently affect video frames [3,4]. Additionally, the simultaneous handling of multiple visual tasks, including object recognition, segmentation, and temporal alignment, makes designing an effective video instance segmentation system capable of delivering high-quality results a significant challenge. Driven by the demand for intelligent sensing for visual perception, how to design lightweight and real-time segmentation systems in resource-constrained environments is an important direction of current research.

Earlier VIS approaches are grouped into four distinct types, determined by the method used to produce instance sequences [5]. The first class of mainstream methods follows the detection-tracking paradigm [6], which adds tracking branches to the image instance segmentation model, detects and segments each frame for each frame, and associates the frames utilizing classification or re-recognition to achieve frame-by-frame instance tracking of the video. The second class of mainstream methods is Clip-match, which divides the whole video into multiple overlapping segments and performs independent video instance segmentation for each segment employing mask propagation [7] and spatiotemporal embedding [8]; ultimately, the instance sequence is generated by merging neighboring segments. Both methodologies rely on a two-stage process to form the full sequence. Initially, several partial sequences (such as individual frames or segments) are derived from the video. These fragments are subsequently combined through techniques like tracking or correspondence matching, resulting in a unified sequence. Nonetheless, these methods are prone to errors accumulating during the process of combining sequences, particularly in scenarios involving rapid movement or object occlusion. To tackle these challenges, a third major category of methods, known as Propose–Reduce, has been introduced [9]. This approach begins by selecting keyframes, uses them to propagate instance segmentation outcomes across the video to form instance sequences, and subsequently eliminates duplicate sequences for identical objects. By avoiding the process of integrating fragmented sequences, this strategy achieves greater stability and reliability, especially in scenarios where several keyframes are utilized for analysis. Nevertheless, all three types of methods rely on intricate heuristic strategies to establish associations between instances or involve several stages to construct instance sequences, resulting in significant computational overhead and high time complexity [5]. The fourth category of mainstream methods involves the use of Transformer [10]. VisTR [11] is the first to apply Transformer architecture to video instance segmentation, processing multiple frames as input. It frames the VIS challenge as an end-to-end parallel sequence decoding task, directly producing a sequence of masked instances across the video. The core of this approach is a novel, efficient strategy for instance sequence matching and segmentation, which ensures comprehensive supervision and segmentation of the instance sequences at the sequence level. However, VisTR is limited by a predefined number of input queries, which are determined by the video’s duration and the maximum possible number of objects. Each query is mapped to an individual object in every frame, and the query mechanism itself is both intricate and somewhat redundant. Subsequently, IFC [12] further improved the performance and efficiency of VisTR by introducing an inter-frame communication mechanism within the Transformer encoder. Specifically, IFC constructed a memory token for the features of the previous frame and fused this feature token with the current frame features, incorporating temporal information to enhance the model’s ability to capture dynamic changes. Although this method avoids reducing spatiotemporal features into a one-dimensional representation, it still flattens the spatiotemporal characteristics of the Transformer decoder. SeqFormer [13] further improved the performance of IFC by considering that the 1D temporal and 2D spatial domains of instances have different properties and should be handled differently; the attention mechanism is executed independently for each frame, maintaining the spatiotemporal dimension of the features instead of flattening them into a single dimension, which provides a large improvement in segmentation accuracy. Nevertheless, the inefficiencies in the backbone feature extraction network, coupled with the computational burden of the attention mechanism, result in a 34.8% reduction in the frames per second (FPS) of SeqFormer compared to IFC (using ResNet-50 as the backbone, for instance). This makes the system considerably slower when processing high-resolution images.

To address the challenges posed by long training times, massive data sizes, high computational costs, poor generalization, and demanding hardware requirements in current video instance segmentation methods, this research introduces a streamlined and efficient framework named DCFA-LVIS (Dual-Channel and Frequency-Aware Approach for Lightweight Video Instance Segmentation). The key contributions of this study are outlined below:

1. The DCFA-LVIS model introduced in this work offers a streamlined and effective end-to-end system that processes an input video, regardless of its length, and simultaneously generates the classification, frame sequence, and mask sequence. This approach eliminates the need for separate tracking components or manual post-processing, effectively preventing the risk of error accumulation.
2. The DCFA-LVIS approach demonstrated in this study shows notable improvements when evaluated on the YouTube-VIS 2019 benchmark. These enhancements are evident across various metrics, including average accuracy (AP), average completeness (AR), model parameter count, and detection rate (FPS).
3. The DCEResNet backbone in the DCFA-LVIS model effectively captures the temporal features of the video using a dual-channel feature enhancement mechanism. At the same time, it controls the number of parameters and computational complexity.
4. The Dual Frequency Awareness Enhanced Network Module in the DCFA-LVIS model proposed in this paper introduces the Dual Frequency Awareness Transformer Decoder. This decoder employs an innovative instance-tracking mechanism that focuses on the motion trajectories of instances, enabling the learning of rich instance sequence representations. By utilizing frame-level feature queries, the model ensures that the attention mechanism consistently focuses on the same instance across frames, effectively aggregating information across the entire video and enhancing global feature learning. The decoder processes high-frequency and low-frequency information in separate layers, reducing computational complexity, minimizing overhead, and improving speed while lowering memory consumption. This ensures outstanding performance under lightweight conditions.

**Figure 1:**
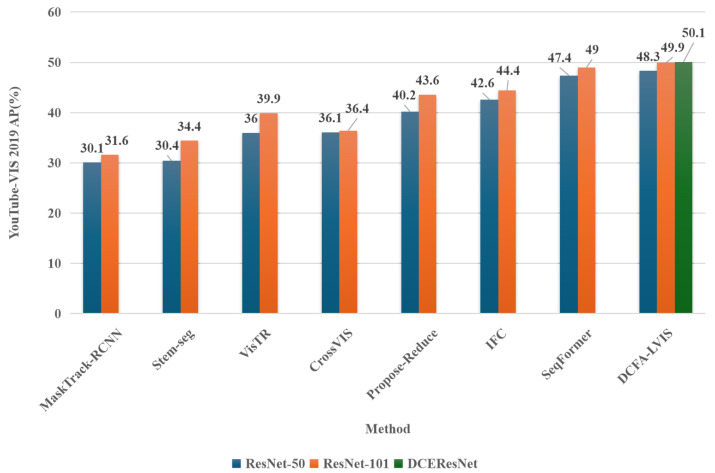
A comparison of the average precision (AP) scores of the leading models in the field of video instance segmentation currently considered state-of-the-art (SOTA).

**Figure 2:**
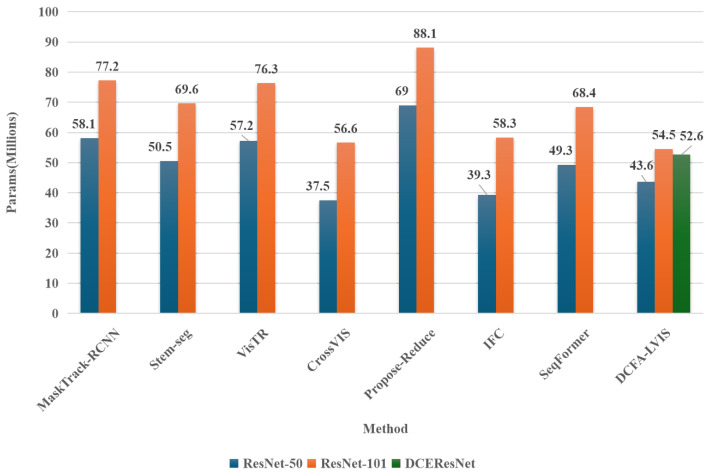
A comparison of the parameter counts (Params) of the leading model in the current state-of-the-art (SOTA) video instance segmentation algorithms.

Figure 1 and Figure 2 present comparative histograms depicting the AP scores and the parameter counts of various video instance segmentation models using ResNet-50, ResNet-101, and DCEResNet (the backbone introduced in this study) as their core networks. All results are derived from a single model configuration and a single inference scale. Among the latest advancements in video instance segmentation, SeqFormer stands out as the current top model, achieving a peak AP of 49% with a parameter count of 68.4 million. The DCFA-LVIS framework introduced in this work achieves a top AP of 50.1%, surpassing the prior approach by 1.1%. Additionally, the parameter count is reduced by 15.8 million, bringing the total to just 52.6 million.

## 2. Related Work

Image instance segmentation refers to a method that enables precise identification and separation of individual objects within an image. It allows for the annotation of each distinct object in the image [14], supporting various computer vision tasks such as object detection [15] and scene interpretation [16]. On the other hand, video instance segmentation involves both recognizing each object in a video and tracking them across frames. This method combines image segmentation techniques with video tracking, posing additional challenges but offering promising potential for future applications [17].

### 2.1. Image Instance Segmentation

Image instance segmentation techniques combine elements of object detection and semantic segmentation, with the primary goal being to categorize every pixel in a given image. This approach allows for the differentiation of individual instances within the same category by utilizing the principles of semantic segmentation to separate them. For a long time, the Mask-RCNN [18] architecture has been the mainstream, which adopts the ResNet-FPN [19] structure as the base network, and its multilayer feature maps help to detect multi-scale objects and small objects. The Mask-RCNN adds a mask branch on top of classification and regression and utilizes a fully convolutional network structure for pixel-level category prediction, which achieves a good segmentation effect. In recent years, one-stage models have gradually become an excellent framework for instance segmentation. Chen H et al. introduced BlendMask [20], a method that combines the concepts behind Mask-RCNN and YOLACT [21]. It incorporates both Top-Down and Bottom-Up strategies [22]. Specifically, it enhances lower-level features by transferring information from the high-level semantic features downwards (Top-Down), while also collecting and consolidating details from the lower-level features upwards to build rich semantic representations at the higher levels (Bottom-Up). By incorporating an attention mechanism into FCOS [23], a novel module that utilizes attention guidance is introduced to extract global feature representations. The underlying module of BlendMask can output higher resolution masks, and when segmenting, not only the results of the target detection are used, but also the information of the FPNs is fused in, improving the segmentation accuracy. He K and his team introduced the PointRend method [24], a novel approach designed to enhance image segmentation. This technique specializes in refining the segmentation of object boundaries, allowing for improved performance in areas where distinguishing the outline of objects proves challenging. PointRend solves the problem of how to compute the masks quickly, reduces the consumption of resources, improves the segmentation accuracy, and has scalability advantages. It revisits the image segmentation problem and breaks new ground by proposing new solutions from the perspective of image rendering. Suresha M [25] et al. found that PointRend has good segmentation results for occlusion-intensive motion videos. QueryInst [26] is an advanced instance segmentation approach that operates in an end-to-end fashion, incorporating query-driven object detection mechanisms and six parallel dynamic masking components. This method demonstrates exceptional performance in the domain of video instance segmentation, outperforming all other online video segmentation techniques. It achieves an optimal trade-off between processing speed and segmentation precision.

### 2.2. Video Instance Segmentation

In video object segmentation, the outcome is not dependent on any predefined category, and as a result, it is not possible to directly acquire the category details of the target object. Unlike it, Video Instance Segmentation (VIS) involves predefined semantic categories, and the network needs to output the corresponding segmentation results and semantic categories, which is similar to Image Instance Segmentation. The MaskTrack R-CNN method [2] successfully adapts the concept of image-based instance segmentation for use in video analysis. It incorporates a novel tracking component built on Mask R-CNN [18], which allows for the identification and tracking of objects across consecutive frames. The SipMask method [27] introduces an efficient spatial preservation (SP) module that produces independent spatial coefficients for different sub-regions within each bounding box. This enhances mask predictions and enables more precise modeling of spatial relationships. CompFeat [28] leverages both temporal and spatial context to enhance features at both the frame and object levels, resulting in substantial improvements in feature differentiation and tracking performance. This approach removes the uncertainty that arose from relying solely on single-frame features in the past for tasks such as detection, segmentation, and tracking. CrossVIS [29] introduces a novel cross-learning approach that enhances model performance by leveraging instance-specific features from the current frame to pinpoint corresponding instances in other frames at the pixel level. This method enables efficient learning of the relationships between instances and pixels across frames, while also minimizing the model’s parameter count without sacrificing accuracy. Stem-seg [8] models the entire video clip and uses 3D convolution to process feature embeddings containing spatiotemporal information with a straightforward structure.MaskProp [7] and Propose–Reduce [9] use mask propagation to improve segmentation accuracy, but the huge amount of computation limits its practicality. In addition, three frequency-aware methods provide valuable insights for VIS. The decomposition model by Xu et al. [30] lays the groundwork for robust feature extraction under challenging conditions, such as low-light environments. Chen et al.’s [31] feature fusion module improves feature consistency and boundary precision, crucial for precise segmentation. Lastly, Warren et al.’s [32] motion-guided edge refinement approach informs strategies for maintaining temporal consistency and accurate edge segmentation in VIS.

### 2.3. Transformers

Initially introduced for tasks related to machine translation [11], the Transformer model’s primary concept revolves around utilizing an attention mechanism to process and encode sequences of natural language text. In contrast to convolutional models, the self-attention framework employed by Transformer architectures provides a comprehensive perspective and excels at capturing long-range dependencies in sequential data. This has led to its extensive adoption in various natural language processing applications, including tasks like translation [33], text classification [34], answering questions [35], and generating text [36]. More recently, Transformers have demonstrated their effectiveness in numerous visual tasks, including object detection [37], tracking [38], segmentation [39], and video analysis [40].

VIT [41] pioneered the application of Transformer models in visual data processing by fragmenting images into smaller segments to facilitate effective modeling. This method demonstrated a performance level on par with traditional deep learning structures like convolutional neural networks. DETR [42] redefined the detection process by employing a Transformer-based structure capable of producing location and classification outputs directly. By bypassing complex traditional processes such as redundant filtering, coordinate corrections, and confidence scoring adjustments, this method significantly simplifies object identification tasks. Deformable DETR [43] solves the problems of DETR in small target detection and convergence speed through local attention mechanism and multi-scale feature mapping and obtains better performance. However, it requires sampling the position of each pixel, which increases the computational complexity and leads to an increase in the time cost of training and inference. VisTR [11] represents the pioneering application of Transformer architectures in video instance segmentation. Leveraging hierarchical supervision and segmentation across sequences allows for integrated processing of instance data. Nevertheless, the requirement to learn separate embeddings for every instance in each frame imposes limitations on its scalability, particularly for extended video sequences and intricate environments. IFC [12] incorporates mechanisms for exchanging information across frames within the encoder, offering improvements in both accuracy and speed compared to VisTR. Nevertheless, its decoder processes the feature representations in a linearized form, disregarding the critical spatial layouts and temporal patterns embedded in the feature data. SeqFormer [13] borrows ideas from VisTR and IFC and proposes to use an instance Query to capture the temporal sequence of instances in a video and independently perform an internal attention mechanism for each frame, significantly improving the model performance. However, the SeqFormer model is structurally complex, computationally and temporally costly, inefficient on high-resolution images, and has a long training time. As a result, developing an algorithm for segmenting video instances that achieves both high-quality results and efficient performance remains a significant challenge.

## 3. Methodology

**Figure 3:**
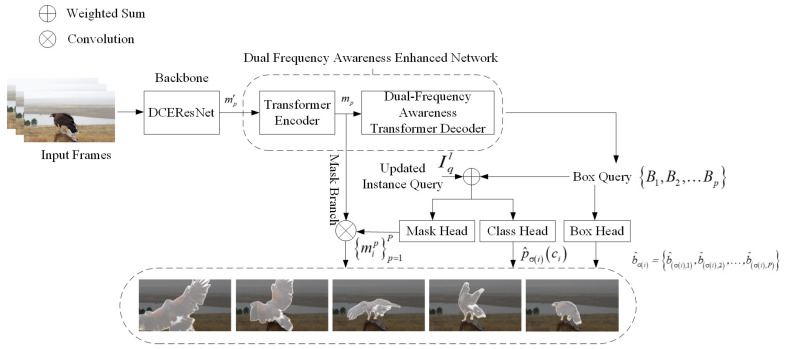
The comprehensive framework of DCFA-LVIS.

To better leverage temporal characteristics inherent in video data while simplifying the model structure, this paper introduces a dual-channel frequency-aware lightweight video instance segmentation method, referred to as DCFA-LVIS. This approach aims to enhance segmentation efficiency without compromising accuracy, as illustrated in Figure 3. Initially, the DCEResNet backbone network processes each video frame individually to extract its corresponding feature representations. These representations are then combined by dynamically fine-tuning convolutional kernel coefficients through the dual-channel feature enhancement mechanism, ultimately producing updated feature maps. The features are then passed into the Dual Frequency Awareness Enhanced network module. Here, the Dual-Frequency Awareness Transformer Decoder assigns shared instance queries to each frame, enabling independent object localization and feature extraction. This ensures the accuracy of the extracted information. Next, the features from all video frames are consolidated into a unified global representation, which helps with object classification and determines the dynamic convolution parameters for segmentation tasks. To avoid the computational overhead associated with the frame-by-frame execution of the attention mechanism, a dual-frequency enhancement mechanism is proposed, which divides the self-attention head into high-frequency and low-frequency attention layers: the high-frequency layer focuses on capturing the local details, while the low-frequency layer focuses on the global structure. The grouping strategy effectively minimizes both the parameter count and computational demands by partitioning the learning parameters into smaller-scale matrices. Subsequently, low-frequency and high-frequency components are merged and forwarded to the following layers, enabling streamlined and efficient instance segmentation in video data.

### 3.1. The Comprehensive Framework of DCFA-LVIS

The design of the DCFA-LVIS network is depicted in Figure 3. Its structure includes three primary modules, among which the Dual-Channel Enhancement ResNet (DCEResNet) plays a critical role in extracting feature representations from each video frame independently. The second component, the Dual Frequency Awareness Enhanced Network, is designed to identify sequential instances and construct video-level representations for them. The third part, known as the output head, handles tasks such as categorizing instances, segmenting instance sequences, and predicting boundary frames for bracketing.

#### 3.1.1. Backbone: DCEResNet

Video instance segmentation usually requires deep feature representations to handle complex visual scenes. Meanwhile, video data usually have higher dimensionality and complexity because it contains temporal dimension. Thus, balancing dimensionality and complexity highlights the need to enhance the processing efficiency of deep neural networks for sequential data. This requires effectively leveraging video temporal characteristics while managing both the parameter count and computational demands.

Convolutional operations are core components in neural networks. In traditional convolutional operations, the weights of the convolutional kernel are predefined and statically fixed, and the convolutional kernel processes the inputs in the same way regardless of changes in the input data. However, in practice, different regions of input data often contain different feature patterns, which makes the static convolution kernel’s performance limited when processing diverse data. Therefore, introducing the mechanism of adaptive convolutional weights can enable the convolutional kernel to dynamically adjust according to the different input features, thus adapting to the changes in data features more effectively. Following this concept, DCFA-LVIS employs DCEResNet as its backbone for extracting features and incorporates a dual-channel mechanism aimed at enhancing the network’s ability to learn and represent features effectively. This mechanism divides the input feature map into two parallel processing paths. In Channel 1, global information is utilized to adjust the weights dynamically between channels, enabling the implementation of a channel attention mechanism. This allows the network to adaptively assign importance to features from different channels and generate the corresponding convolutional weights. Channel 2 focuses on capturing local spatial data, and the outputs from both channels are merged during the convolution process. This design results in a more comprehensive and precise feature representation by integrating global context with detailed local features.

**Figure 4:**
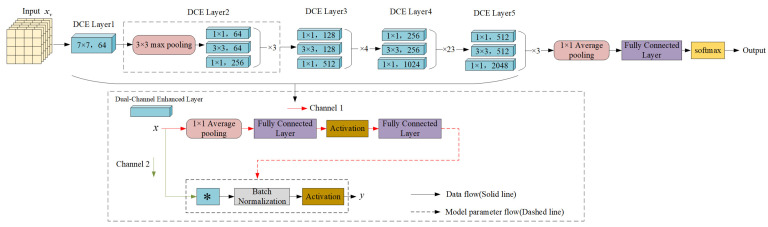
Structure Diagram of DCEResNet: A Dual-Channel Enhanced ResNet Network.

The structure of the DCEResNet network is illustrated in Figure 4. For an input video $x_{v}\in R^{P\times 3\times H\times W}$, where P represents the number of frames, and the video consists of 3 color channels; the backbone network DCEResNet processes each frame of the video independently to extract its feature representations. Here, H refers to the height, or the number of vertical pixels in a frame, while W denotes the width or the number of horizontal pixels.

As the video frame moves through the dual-channel feature enhancement backbone network, it first encounters a feature enhancement layer (DCELayer1) with a convolutional filter size of 7 × 7 and 64 output channels. This layer efficiently captures a broader spectrum of features, expands the receptive field, minimizes image detail loss, and supplies richer preliminary data for further feature extraction. To begin with, the 1 × 1 dual-channel feature enhancement layer reduces the number of input channels to 64, which lowers both the computational workload and the parameter count, improving the model’s overall performance. Next, the 3 × 3 dual-channel feature enhancement layer performs convolution on the reduced-dimension feature maps. This extracts more spatial information and enhances the ability to capture nonlinear features while preserving important structural details from the input image. Finally, the 1 × 1 dual-channel feature enhancement layer increases the number of channels to 256. This restores a higher-dimensional feature representation, creating more space for the subsequent feature fusion. Furthermore, to maintain smooth information transfer across the deep network, the dual-channel feature enhancement block incorporates a skip connection, similar to those used in ResNet. This connection helps stabilize the gradient during backpropagation by directly linking the input and output. It reduces the vanishing gradient problem and also lowers the computational load. This approach not only enhances the efficiency of the model’s training process but also ensures that crucial semantic details are preserved. The subsequent dual-channel feature enhancement layers (DCELayer3 to DCELayer5) are consistent with the design principle of DCELayer2 and are constructed through maximum pooling layers and multiple dual-channel feature enhancement blocks to extract higher-level semantic features layer by layer. As the network deepens, these layers further capture global contextual information while preserving fine-grained spatial details, thus enhancing overall feature representation. The design principle of the dual-channel feature enhancement layer is described in detail below.

The dual-channel feature enhancement layer solves the issue where standard convolution uses the same kernel for all input images. This approach helps prevent a significant increase in the number of parameters. This increases the speed of the network model and also reduces the redundancy of the structure. The core of the dual-channel feature enhancement layer is that its convolution kernel weights are adaptively adjusted, which is implemented as follows:

**Channel Attention Mechanism**  $A\left(x\right)$. Firstly, average pooling is performed on the input feature map $x_{v}\in R^{C\;\times \;H\;\times \;W}$. The pooling operation is performed independently for each channel, compressing the information of the spatial dimension H×W into a single scalar to obtain the channel vector $z\in R^{C}$, which is computed as shown in Equation (1):

(1) $$z_{i}=\frac{1}{H\times W}\sum_{h=1}^{H}\sum_{w=1}^{W}x_{i,h,w}$$

where $z_{i}$ denotes the global mean of the ith channel.

The average pooled vector z is passed through a fully connected layer weight matrix $W_{1}\in R^{C/r\;\times \;C}$, where r is the dimensionality reduction rate (usually r=16 or r=8), and the input vector z is linearly transformed to generate a low-dimensional vector, which is computed as shown in Equation (2):

(2) $$z^{\prime }=W_{1}z+b_{1}$$

The output $z^{\prime }\in R^{C/r}$, reducing the computational effort through dimensionality reduction. where $b_{1}\in R^{d}$ represents the bias vector that allows for a translation adjustment of the linearly transformed result $W_{1}z$, increasing the flexibility of the model and ensuring a non-zero output even when the input is zero.

The nonlinear activation of the vector $z^{\prime }$ after dimensionality reduction is usually performed using the ReLU function, which is computed as shown in Equation (3):

(3) $$\begin{array}{c} z^{\prime \prime }=\mathrm{ReLU}\left(z^{\prime }\right)\\ =\left\{\begin{array}{c} z^{\prime },z>0\\ 0,z⩽0 \end{array}\right. \end{array}$$

The activated vector $z^{\prime \prime }$ is then passed through another fully connected layer weight matrix $W_{2}\in R^{C\;\times \;C/r}$, which brings the number of channels back to the original value C, and this operation is detailed in Equation (4):

(4) $$\hat{z}=W_{2}z^{\prime \prime }+b_{2}$$

Here $\hat{z}\in R^{C}$, represents the weight corresponding to each channel. $b_{2}$ is the bias vector.

To constrain the output weights within a range of 0 to 1, $\hat{z}$ is finally normalized by a Sigmoid function, calculated as shown in Equation (5):

(5) $$\alpha_{i,i}\left(x\right)=\mathrm{Sigmoid}\left({\hat{z}}_{i}\right)$$

The obtained $\alpha_{i,i}\left(x\right)$ is the diagonal element in the channel attention matrix $A\left(x\right)$.

$A\left(x\right)$ represents a matrix where only the diagonal elements are non-zero, with each diagonal entry, $\alpha_{i,i}\left(x\right)$, being a function of the input x. By multiplying $K_{\mathrm{avg}}$ with $A\left(x\right)$, dynamic weighting of individual channels is achieved. Here, $K_{\mathrm{avg}}=\frac{1}{S}\sum_{s=1}^{S}K_{s}$ represents the mean convolution kernel ($K_{s}$ comprises s static convolution kernels, $s\in \left\{1,\ldots ,S\right\}$) and is a C×C matrix. Channel weights can be adjusted before convolution to enhance the adaptability of feature extraction. The formulation of the channel attention matrix $A\left(x\right)$ can be expressed as in Equation (6):

(6) $$A\left(x\right)=\mathrm{diag}\left(\alpha_{1,1}\left(x\right),\alpha_{2,2}\left(x\right),\ldots ,\alpha_{C,C}\left(x\right)\right)$$

**Adaptive Channel Fusion**  $M\Psi \left(x\right)N^{T}$. The input feature x first undergoes the transposition of N, denoted as $N^{T}x$, which reduces the original C-dimensional features to an L-dimensional latent space. This phase aims to decrease both the parameter count and the computational demand. Subsequently, the resulting low-dimensional feature $N^{T}x\in R^{L}$ passes through an adaptive fusion matrix $\Psi \left(x\right)$ (for the generation process of $\Psi \left(x\right)$, please refer to $A\left(x\right)$), which captures the relationships between different channels. Here, $\Psi \left(x\right)$ is an L×L matrix, where each element $\varphi_{i,j}\left(x\right)$ is a function of the input x, representing the adaptive interaction between channels. Finally, the matrix M is used to project the fused features back to the C-dimensional space, expressed as $M\Psi \left(x\right)N^{T}x$, thereby generating convolutional weights that adapt to the input features.

**Generate adaptive convolutional weights**  $K\left(x\right)$. By combining the above two parts, the complete adaptive convolutional weight generation Formula (7) is obtained:

(7) $$\begin{array}{cc} K(x) & =A\left(x\right)K_{\mathrm{avg}}+\sum_{i=1}^{L}\sum_{j=1}^{L}m_{i}\varphi_{i,j}\left(x\right)n_{j}^{T}\\ \; & =A(x)K_{\mathrm{avg}}+M\Psi \left(x\right)N^{T} \end{array}$$

This formula indicates that the adaptive convolutional weights consist of two components: the static convolutional weights $A\left(x\right)K_{\mathrm{avg}}$, which are adaptively adjusted based on the input, and the adaptive weights $M\Psi \left(x\right)N^{T}$, derived from dimensionality reduction, dynamic fusion, and dimensionality restoration. Specifically, N is a C×L matrix, where $n_{j}$ represents the j-th column vector of N, which serves to map the input channels from C down to L, effectively reducing dimensionality. Similarly, M is a C×L matrix, where $m_{i}$ denotes the i-th column vector of M, which restores the channel count from L to C for dimensionality restoration. The resulting L channels are adaptively fused by $\Psi (x)\in R^{L\times L}$, aggregating channel information to produce the adaptively adjusted $K\left(x\right)$, which represents the convolutional weights derived from the input x. The dual-channel feature enhancement layer significantly reduces the number of static parameters (i.e., the parameters L and C in M or N), resulting in a more compact model.

Next, this paper examines how the dual-channel feature enhancement layer helps reduce the number of parameters. It starts with an analysis of parameter complexity. According to Equation (7), the parameter count for the static convolution kernel $K_{\mathrm{avg}}$ is given by C×C. The channel attention matrix $A\left(x\right)$ is diagonal, so its parameter count is C (i.e., the number of elements on the diagonal). The parameter count of matrices M and N is C×L, and the parameter count of the adaptive fusion matrix $\Psi \left(x\right)$ is L×L. The number of parameters required to generate $A\left(x\right)$ and $\Psi \left(x\right)$ through channel one (via the fully connected layer) is $\left(2C+L^{2}\right)C/r$, where r is the dimensionality reduction ratio of the fully connected layer. Therefore, the total parameter count of the dual-channel feature enhancement layer is given by Equation (8):

(8) $$\begin{array}{cc} {\mathrm{Param}s}_{\mathrm{DCE}} & =C^{2}+2\mathrm{CL}+L^{2}+\frac{(2C+L^{2})C}{r}\\ \; & \approx \left(1+\frac{3}{r}\right)C^{2}+2C\sqrt{C} \end{array}$$

For a standard convolutional layer, the total number of parameters can be expressed by Equation (9):

(9) $${\mathrm{Params}}_{\mathrm{conv}}=C^{2}\times k\times k$$

A comparative analysis of Equations (8) and (9) reveals that the parameter complexity of standard convolution is primarily influenced by the dimensions of the kernel (k×k) as well as the number of input and output channels C. For small convolution kernels with k=3 or k=5, the number of parameters still increases significantly. In contrast, in the dual-channel feature enhancement layer, where L≪C, the total parameter count is significantly lower than that of the standard convolution layer after applying the matrices M, N, and the reduced-dimensional dynamic fusion matrix $\Psi \left(x\right)$. This advantage is particularly evident when k is large or when the quantities of input and output channels C are large, making the dual-channel feature enhancement layer more efficient.

**Figure 5:**
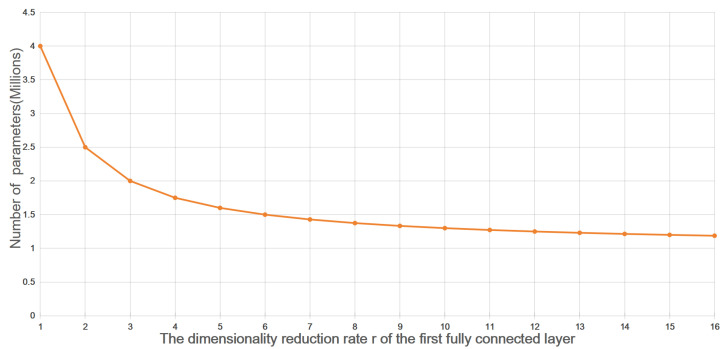
Dual-channel feature enhancement layer parameter count complexity trend plot.

As shown in Figure 5, an increase in the dimensionality reduction ratio r of the initial fully connected layer results in a gradual decrease in the parameter complexity of the dual-channel feature enhancement layer. When r=16 is chosen, the complexity is approximately $1\frac{3}{16}C^{2}$, which is much smaller than the complexity of the 3×3 standard convolution layer, $9C^{2}$. Therefore, the dual-channel feature enhancement mechanism offers a clear advantage in video instance segmentation tasks by improving computational efficiency. Furthermore, video instance segmentation needs to account for the temporal changes in objects over time. The dual-channel feature enhancement layer provides finer control over the convolutional kernel weights. This allows it to adapt to changes in the position and shape of objects across different frames, helping to better capture the trajectories and movements of object instances.

#### 3.1.2. Dual Frequency Awareness Enhanced Network Module

**Figure 6:**
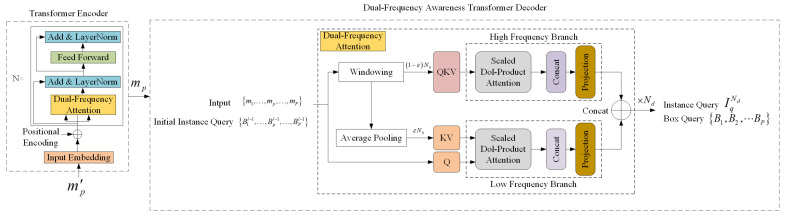
Dual-frequency-enhanced network structure diagram.

Traditional Transformer-based video instance segmentation methods, such as VisTR [11], usually use frame-level instance queries. The number of instance queries is a hard-coded setting based on the video length and the maximum number of instances. This approach can lead to excessive computational overhead when dealing with long sequences. Additionally, although the attention mechanism applied independently to each frame can capture intra-frame features, it neglects the spatiotemporal continuity between video frames. Therefore, to more efficiently model instance changes and cross-frame associations in videos, this paper proposes the Dual Frequency Awareness Enhanced Network, with the overall network architecture illustrated in Figure 6.

**Transformer Encoder**. The output of the backbone network’s feature map is processed through a 1×1 enhancement layer, which utilizes the dual-channel feature boosting method discussed in Section 3.1.1. This layer reduces the dimensionality of the channels to 256, resulting in the creation of a refined feature map ${\left\{{m^{\prime }}_{p}\right\}}_{p=1}^{P}$, ${m^{\prime }}_{p}\in R^{C\times H^{\prime }\times W^{\prime }},p\in \left[1,P\right]$. In this case, C denotes the total number of channels, while $H^{\prime }$ refers to the vertical size of the newly produced feature map (essentially the number of pixels along the height of the video frame). Similarly, $W^{\prime }$ indicates the horizontal size of the newly produced feature map (i.e., the pixel count along the width of the video frame). As shown in Figure 6, within the dual-frequency enhancement framework, the input data are first processed through a trainable embedding layer. This transforms the data into high-dimensional vector representations. To add positional context to the embedded vectors, positional encoding is applied. This helps the Transformer model better capture the sequential dependencies within the input data. Due to the Transformer’s inability to recognize sequence order on its own, additional positional data must be incorporated. After embedding static positional encodings, the encoder employs a dual-frequency attention mechanism, processing the feature map and producing an output map ${\left\{m_{p}\right\}}_{p=1}^{P}$ that retains the original resolution of the input ${m^{\prime }}_{p}$. In the diagram, N denotes the total number of layers in the encoder structure. Residual connections and normalization layers (Add and LayerNorm) are employed to mitigate the issue of vanishing gradients that arise in deeper networks, which help maintain the stability of feature distributions. The feedforward component typically consists of a series of linear transformations followed by activation functions. This allows each input feature to be processed independently and mapped nonlinearly, improving the model’s ability to capture complex relationships. To apply the attention mechanism independently to each frame, this approach keeps the original structure of the feature map. It preserves both the spatial layout and temporal properties, rather than compressing them into a single representation.

**Dual-Frequency Awareness Transformer Decoder**. Within the decoder part of this study, a set number of trainable embeddings is introduced to extract identical instance features from each frame. These are referred to as instance queries. Given the variations in the appearance and positioning of instances, it is essential for the model to concentrate on accurately identifying the spatial coordinates within each frame. To achieve this, this paper decomposes the instance sequence into a sequence of box queries for P specific frames, which serve as anchors to retrieve and locate the corresponding frame features. The input video sequence is represented by $M={\left\{m_{p}\right\}}_{p=1}^{P}$, where $m_{p}$ refers to the feature map corresponding to the p-th frame.

In the initial layer of the decoder, a portion of the input sequence is used as the initial instance Query $I_{q}\in R^{C}$, enabling the independent extraction of instance features from the feature map of each individual frame, as shown in Equation (10):

(10) $$\begin{array}{cc} B_{p}^{1} & =\mathrm{DFEAttn}\left(I_{q},m_{p}\right),\\ \; & =\left[\mathrm{HF}\left(I_{q},m_{p}\right)\&\mathrm{LF}\left(I_{q},m_{p}\right)\right],\\ \; & =\mathrm{Softmax}\left(\frac{Q_{h}{K_{h}}^{T}}{\sqrt{d}}\right)V_{h}\\ \; & \;\&\mathrm{Softmax}\left(\frac{Q_{l}{\left(\mathrm{AvgPool}\left(K_{l}\right)\right)}^{T}}{\sqrt{d}}\right)\mathrm{AvgPool}\left(V_{l}\right) \end{array}$$

Here, $B_{p}^{1}\in R^{C}$ represents the box query sequence for the p-th frame from the first encoder layer, and $m_{p}$ represents the input feature map. DFEAttn refers to the Dual-Frequency-Enhanced Attention mechanism, with $\mathrm{HF}\left(I_{q},m_{p}\right)$ representing the high-frequency attention branch and $\mathrm{LF}\left(I_{q},m_{p}\right)$ representing the low-frequency attention branch. $Q_{h}$, $K_{h}$, and $V_{h}$ are the query, key, and value matrices extracted from the original feature map, representing the local window features of the high-frequency branch. $\frac{Q_{h}K_{h}^{T}}{\sqrt{d}}$ is the scaled dot product between the query and key, and d is the scaling dimension. Attention weights are obtained through Softmax and then multiplied by $V_{h}$ to obtain the high-frequency weighted values. $Q_{l}$, $K_{l}$, and $V_{l}$ represent matrices associated with the query, key, and value, respectively, which are obtained from the feature map after downsampling. These matrices reflect the low-frequency components extracted through average pooling. Attention weights are generated from the scaled dot product of $Q_{l}$ and $\mathrm{AvgPool}\left(K_{l}\right)$, and applied to $\mathrm{AvgPool}\left(V_{l}\right)$ to obtain the low-frequency weighted values. The $\left[\&\right]$ denotes concatenation, where high-frequency features come first, followed by low-frequency features.

In the decoder layer indexed by l (where l>1), the output $B_{p}^{l-1}$ of the previous layer is used as input as shown in Equation (11):

(11) $$\begin{array}{cc} B_{p}^{l} & =\mathrm{DFEAttn}\left(B_{p}^{l-1},m_{p}\right),\\ \; & =\left[\mathrm{HF}\left(B_{p}^{l-1},m_{p}\right)\&\mathrm{LF}\left(B_{p}^{l-1},m_{p}\right)\right],\\ \; & =\mathrm{Softmax}\left(\frac{Q_{h}^{\left(l\right)}{K_{h}^{\left(l\right)}}^{T}}{\sqrt{d}}\right)V_{h}^{\left(l\right)}\\ \; & \;\&\mathrm{Softmax}\left(\frac{Q_{l}^{\left(l\right)}{\left(\mathrm{AvgPool}\left(K_{l}^{\left(l\right)}\right)\right)}^{T}}{\sqrt{d}}\right)\mathrm{AvgPool}\left(V_{l}^{\left(l\right)}\right) \end{array}$$

Here, $Q_{h}^{\left(l\right)}$, $K_{h}^{\left(l\right)}$, and $V_{h}^{\left(l\right)}$ represent the high-frequency query, key, and value matrices for the l-th layer, while $Q_{l}^{\left(l\right)}$, $K_{l}^{\left(l\right)}$, and $V_{l}^{\left(l\right)}$ denote the corresponding low-frequency matrices for the same layer. Each layer recursively applies high-frequency and low-frequency attention mechanisms, allowing the model to effectively extract detailed and overarching features across different levels of the network. The instance queries assign weights to the box sequences output by each decoder layer and aggregate the temporal features. These weights are learned end-to-end during the box embedding process. This process is expressed in Equation (12), where FC denotes a fully connected layer, and $I_{q}^{l}$ represents the instance query sequence obtained from the l-th decoder layer:

(12) $$I_{q}^{l}=\frac{\sum_{p=1}^{P}B_{p}^{l}\times \mathrm{FC}\left(B_{p}^{l}\right)}{\sum_{p=1}^{P}\mathrm{FC}\left(B_{p}^{l}\right)}+I_{q}^{l-1}.$$

After the $N_{d}$-th decoder layer, an instance query sequence $I_{q}^{N_{d}}$ and P box query sequences ${\left\{B_{p}^{N_{d}}\right\}}_{p=1}^{P}$ for each instance are derived. The instance query serves as a shared representation of the video-level instance. Meanwhile, the box queries hold the spatial information needed to predict the bounding boxes for each frame. The instance sequence $I_{q}^{N_{d}}$ and box query sequence ${\left\{B_{p}^{N_{d}}\right\}}_{p=1}^{P}$ output by the final decoder layer are designated as the output instance embedding and box embedding ${\left\{BE_{p}\right\}}_{p=1}^{P}$,$BE_{p}\in R^{N\times d}$, respectively.

#### 3.1.3. Output Heads Setting

Figure 3 illustrates that, in this study, an additional output layer is integrated into the dual-frequency enhancement network module. This layer is made up of three distinct parts: one for classification, one for bounding boxes, and one for mask generation. The classification component produces output labels via a linear transformation. The Transformer decoder denotes the number of instances as $\sigma \left(i\right),i\in Z$, representing the i-th instance. Let the class of an instance be $c_{i}$, and the classification head outputs the class probability ${\hat{p}}_{\sigma \left(i\right)}\left(c_{i}\right)$ for $c_{i}$, where $c_{i}$ can be ∅.

The box head consists of a three-layer feedforward network (FFN) that utilizes ReLU for activation, followed by a linear projection stage. For the p-th frame’s box embedding ($BE_{p}$), it estimates the normalized values for the center’s location, height, and width. Thus, for the instance labeled as $\sigma \left(i\right)$, the sequence of predicted frames is represented as ${\hat{b}}_{\sigma \left(i\right)}=\left\{{\hat{b}}_{\left(\sigma \left(i\right),1\right)},{\hat{b}}_{\left(\sigma \left(i\right),2\right)},\ldots ,{\hat{b}}_{\left(\sigma \left(i\right),p\right)}\right\}$, where ${\hat{b}}_{\left(\sigma \left(i\right),p\right)}$ corresponds to the forecasted frame for that instance.

For the mask head, the dual-channel feature enhancement layer can be utilized as the mask head. The decoder’s output instance embeddings encapsulate instance information across all frames, making it a more robust instance representation compared to previous approaches. These embeddings, for instance, are leveraged to quickly construct the full sequence of masks. Initially, a neural network with three layers processes the instance embedding to produce the mask head parameter $\omega_{i}$ for the $\sigma \left(i\right)$-th instance. Identical instances across various frames share the same mask head parameters, which optimizes the segmentation process. Every convolutional layer consists of 8 channels, and ReLU is used as the activation function for all layers except the final one, where a dual-channel enhancement method is applied. The mask branch is responsible for generating the feature maps required to predict the masks for each instance. It adopts a structure similar to Feature Pyramid Networks (FPNs) and utilizes the Transformer encoder to generate a multi-scale feature map sequence $\left\{{\hat{F}}_{\mathrm{mask}}^{1},{\hat{F}}_{\mathrm{mask}}^{2},\ldots ,{\hat{F}}_{\mathrm{mask}}^{P}\right\}$, where the resolution of each frame is $\frac{1}{8}$ of the input, and each channel is independent, with a total of 8 channels. The feature maps ${\hat{F}}_{\mathrm{mask}}^{p}$ are then concatenated with the relative coordinate maps centered on the corresponding instance prediction box ${\hat{b}}_{\left(\sigma \left(i\right),p\right)}$, providing the spatial information required for mask prediction. This results in high-resolution feature sequences ${\left\{F_{\mathrm{mask}}^{p}\right\}}_{p=1}^{P}$, where $F_{\mathrm{mask}}^{p}\in R^{10\;\times \;\frac{H}{8}\;\times \;\frac{W}{8}}$. Convolutional operations are performed on the high-resolution feature sequences $F_{\mathrm{mask}}^{p}$ for all frames p=1 to P, in order to generate the mask sequence, as outlined in Equation (13):

(13) $${\left\{m_{i}^{p}\right\}}_{p=1}^{P}={\left\{\mathrm{MaskHead}\left(F_{\mathrm{mask}}^{p},\omega_{i}\right)\right\}}_{p=1}^{P}$$

The method presented utilizes MaskHead(·) to process the input feature maps by applying a sequence of three 1×1 convolutions, where the convolution kernels are modified from the mask head parameters $\omega_{i}$. To ensure efficient instance segmentation across individual frames, the same mask head parameters $\omega_{i}$ are applied to instances sharing an identical identity $\sigma \left(i\right)$ across different time steps. Drawing inspiration from the DETR framework [42], DCFA-LVIS integrates both output heads and a Hungarian loss following each layer of the decoder, utilizing them as supplementary losses to guide the model’s training.

### 3.2. Matching of Instance Sequences and Associated Loss Calculation

The proposed approach incorporates additional output layers along with a Hungarian loss function placed after each layer of the decoder. These layers serve as extra loss functions to guide the model during training. The output layers consist of three distinct heads: one for classification, one for bounding boxes, and one for mask generation. At each decoding step, a fixed number, N, of predictions is generated for the video frames. Here, N is chosen to be much greater than the total number of instances present in the video. To train the model, a bipartite matching approach is applied to align the predicted results with the actual ground truth values. Denote the set of ground truth instances for video frames as g, and let ${\hat{g}}_{i}={\left\{{\hat{g}}_{i}\right\}}_{i=1}^{N}$ represent the predicted instances, where i ranges from 1 to N. Each element in the ground truth set, i, can be described as $g_{i}=\left\{c_{i},\left(b_{i,1},b_{i,2},\ldots ,b_{i,P}\right)\right\}$, with $c_{i}$ indicating the category label(where $c_{i}\neq \emptyset$), and $g_{i}$ can be an empty set. The vector $b_{i,p}\in {\left[0,1\right]}^{4}$ represents the positional information such as the center, relative height, and width of the bounding box for ground truth in frame p. Add the corresponding subscript $\sigma \left(i\right)$ to the instance embedding output by the decoder, and the probability of class $c_{i}$ output by the class head is denoted as ${\hat{y}}_{\sigma \left(i\right)}\left(c_{i}\right)$. The index $\hat{\sigma }\left(i\right)$ of the best-matching prediction for the instance is determined using ${\hat{y}}_{\sigma \left(i\right)}\left(c_{i}\right)$ and the estimated bounding box ${\hat{b}}_{\sigma \left(i\right)}$. A cost function for matching pairs is then established between the actual ground truth $g_{i}$ and the predicted instance ${\hat{g}}_{\sigma \left(i\right)}$ identified by index $\sigma \left(i\right)$, as shown in Equation (14):

(14) $$L_{\mathrm{match}}\left(g_{i},{\hat{g}}_{\sigma \left(i\right)}\right)=-{\hat{y}}_{\sigma \left(i\right)}\left(c_{i}\right)+L_{\mathrm{box}}\left(b_{i},{\hat{b}}_{\sigma \left(i\right)}\right)$$

Here, $L_{\mathrm{box}}\left(b_{i},{\hat{b}}_{\sigma }\left(i\right)\right)$ represents the boundary box loss function. Due to the high computational cost of comparing mask-level similarities, Equation (14) does not consider the similarity between predicted and ground truth masks. To find the optimal bipartite matching between the ground truth set and the predicted set, the sequence of N instances $\sigma \in S_{N}$ is determined by searching for the minimum cost, as shown in Equation (15):

(15) $$\hat{\sigma }=\underset{\sigma \in S_{N}}{\arg\min}\sum_{i=1}^{N}L_{\mathrm{match}}\left(g_{i},{\hat{g}}_{\sigma \left(i\right)}\right)$$

Based on the obtained optimal subscript value $\hat{\sigma }\left(i\right)$, following the previous work [10,42], the matching pairs are computed using the Hungarian loss function [44] to obtain the optimal allocation as shown in Equation (16):

(16) $$\begin{array}{cc} \; & L_{\mathrm{Hung}\left(g,\hat{g}\right)}=\sum_{i=1}^{N}[-\log{\hat{y}}_{\hat{\sigma }\left(i\right)}\left(c_{i}\right)\\ \; & \;+I_{\left\{c_{i}\neq \emptyset \right\}}L_{\mathrm{box}}\left(b_{i},{\hat{b}}_{\hat{\sigma }\left(i\right)}\left(i\right)\right)\\ \; & \;+I_{\left\{c_{i}\neq \emptyset \right\}}L_{\mathrm{mask}}\left(k_{i},{\hat{k}}_{\hat{\sigma }\left(i\right)}\left(i\right)\right)] \end{array}$$

where $k_{i}$ represents the true mask value and ${\hat{k}}_{\hat{\sigma }}\left(i\right)$ represents the predicted mask value; $L_{\mathrm{box}}\left(b_{i},{\hat{b}}_{\hat{\sigma }}\left(i\right)\right)$ stands for the loss function that calculates the bounding box coordinates, whereas $L_{\mathrm{mask}}\left(k_{i},{\hat{k}}_{\hat{\sigma }}\left(i\right)\right)$ pertains to the loss function that evaluates the mask sequence.

This paper computes $L_{\mathrm{box}}$ using a linear combination of Smooth $L_{1}$ loss [45] and CIoU loss [46], as shown in Equation (17):

(17) $$\begin{array}{cc} L_{\mathrm{box}}\left(b_{i},{\hat{b}}_{\sigma \left(i\right)}\right) & =\lambda_{SL_{1}}\left\{\begin{array}{cc} 0.5{\left|b_{i}-{\hat{b}}_{\sigma \left(i\right)}\right|}^{2} & \mathrm{if}\;\left|b_{i}-{\hat{b}}_{\sigma \left(i\right)}\right|<1,\\ \left|b_{i}-{\hat{b}}_{\sigma \left(i\right)}\right|-0.5 & \mathrm{otherwise}, \end{array}\right.\\ \; & \;+\lambda_{\mathrm{ciou}}L_{\mathrm{ciou}}\left(b_{i},{\hat{b}}_{\sigma \left(i\right)}\right) \end{array}$$

In this approach, the hyperparameters $\lambda_{SL_{1}},\lambda_{\mathrm{ciou}}\in R$ are real-valued constants. Due to the fact that the mask sequence ${\left\{k_{i}^{p}\right\}}_{p=1}^{P}$ produced by the mask head has a resolution that is only one-eighth of the video resolution, it leads to a loss in fine-grained details. To address this, the predicted masks are upsampled to a resolution that is one-quarter of the video resolution, while the ground truth masks are downsampled to match this resolution. The mask loss is then computed based on this adjusted resolution. Additionally, $L_{\mathrm{mask}}$ is calculated using a weighted sum of the Dice coefficient [47] and E-Focal loss [48], as presented in Equation (18):

(18) $$\begin{array}{cc} L_{\mathrm{mask}} & =\lambda_{\mathrm{dice}}L_{\mathrm{dice}}+\lambda_{\mathrm{efl}}L_{\mathrm{efl}},\\ \; & =\lambda_{\mathrm{dice}}\left(1-\frac{2\left|X\cap Y\right|}{\left|X\right|+\left|Y\right|}\right)+\lambda_{\mathrm{efl}}\left(-\alpha_{t}{\left(1-p_{t}\right)}^{\gamma^{j}}\log\left(p_{t}\right)\right) \end{array}$$

Here, $\lambda_{\mathrm{dice}},\lambda_{\mathrm{efl}}\in R$ is a hyperparameter. Let X denote the set of predictions, while Y represents the actual ground truth values. The predicted confidence score of the candidate object is denoted by $p_{t}\in \left[0,1\right]$. The parameter $\alpha_{t}$ is used to control the relative importance between positive and negative samples. Additionally, $\gamma^{j}$ serves as a factor that adjusts the attention given to the j-th class. Typically, a higher value of $\gamma^{j}$ is applied to address the substantial imbalance between positive and negative examples in rare classes, whereas a smaller $\gamma^{j}$ is more appropriate for classes with a relatively balanced distribution. The Hungarian loss is determined by first calculating the box loss and the mask loss for each individual frame, and then averaging these losses.

## 4. Experimental Results and Analysis

This section begins with a brief overview of the model’s training process and the datasets employed. To evaluate the effectiveness of the DCFA-LVIS model, its performance is assessed on both datasets, followed by a comparison with recent cutting-edge techniques. Visualization results are also provided to support the analysis. Finally, to confirm the contribution of the proposed module, ablation studies are conducted to highlight the enhancements in model performance.

### 4.1. Datasets and Metrics

This research evaluates the proposed methods using two key datasets: YouTube-VIS 2019 [2] and YouTube-VIS 2021 [49]. The YouTube-VIS 2019 dataset, a pioneering resource for video instance segmentation, consists of 2238 high-definition video clips from YouTube for training, and an additional 302 clips for testing. These videos cover a wide range of objects and scenarios, with each clip labeled with bounding boxes and instance masks across 40 different categories. This dataset provides 131,000 precise instance masks to accurately pinpoint pixel-level locations of different object instances. Object instances in the videos are tagged every five frames, offering enough training data while cutting down on labeling costs. The YouTube-VIS 2021 dataset, which enhances the previous version, aims to increase the difficulty and usefulness of video instance segmentation challenges. It comprises 3859 high-def YouTube video clips and 232,000 instance labels, a significant expansion from the 2019 dataset, featuring more instances and frames. Employing these datasets permits the comprehensive testing of the methods against a broader and more rigorous set of video content, verifying their performance and relevance.

To assess the effectiveness of video instance segmentation, two key performance indicators are typically employed: Average Precision (AP) and Average Recall (AR). AP evaluates the mean precision across a set of instances in a video, each described by a sequence of masks. In order to verify the temporal and spatial consistency of these sequences, calculations based on Intersection over Union (IoU) are performed within the spatio-temporal context. This implies that the model must not only excel in segmenting and categorizing individual frames but also ensure the accurate tracking of masks across the entire sequence. The IoU metric measures the degree of overlap between the predicted and ground truth frames, with higher values reflecting better prediction accuracy. Additionally, we present two specific measures, ${\mathrm{AP}}_{50}$ and ${\mathrm{AP}}_{75}$, which are derived at IoU thresholds of 0.5 and 0.75, respectively. These offer a refined evaluation of the model’s performance under different conditions, enabling a more nuanced assessment of its precision. On the other hand, AR evaluates how well the model classifies instances correctly by calculating the ratio of correct predictions to the total samples, where ${\mathrm{AR}}_{1}$ indicates the accuracy based on the single best match and ${\mathrm{AR}}_{10}$ considers the top 10 matches.

Additionally, this study incorporates parameters (Params) and detection frame rate (FPS) in evaluating the DCFA-LVIS model’s performance. The assessment examines DCFA-LVIS comprehensively using eight metrics to affirm the model’s validity.

### 4.2. Implementation Details

#### 4.2.1. Model Settings

In this work, the DCFA-LVIS model utilizes DCEResNet as its primary network unless stated otherwise. As in [43], the feature extraction process focuses on the final three layers of DCEResNet, denoted as C3, C4, and C5, which correspond to feature maps with strides of 8, 16, and 32, respectively. A convolutional layer with a kernel size of 3 × 3 and a stride of 2 is applied to the output from C5, producing a lower-resolution feature map, C6. For the dual-frequency enhancement component, the window size s is configured to 2, and the segmentation ratio ε is set to 0.9. Within the Transformer architecture, the model incorporates six layers each for both the encoder and decoder, with a hidden size of 256. Additionally, the number of instance queries is fixed at 300.

#### 4.2.2. Training

In this study, the AdamW optimizer [50] is employed, with an initial learning rate set to $2\times 10^{-4}$. The hyperparameters $\beta_{1}$, $\beta_{2}$ are chosen as 0.9 and 0.999, respectively, along with a weight decay of $10^{-4}$. The model first undergoes pre-training on the COCO dataset [51], where only a single frame (P = 1) is used. Following this, the DCF-LVIS model is trained on the YouTube-VIS dataset, using five frames (P = 5) from each video, with the pre-trained weights utilized for the training.

In order to reduce overfitting resulting from the limited number of training images in YouTube-VIS, this study supplements the dataset with 80,000 extra images sourced from COCO, as outlined in earlier studies [8,9]. Specifically, in this paper, only images related to the 20 overlapping categories in the COCO dataset and the YouTube-VIS dataset are selected, and they are augmented with enhancement processes, including a ±10-degree rotation, to generate a pseudo-video dataset containing five frames. The model was then trained in this paper for a period of 12 cycles on this hybrid dataset, which consists of both COCO and video datasets. Throughout training, the model employs a learning rate decay strategy, adjusting the rate by 0.1 at the 6th and 10th iterations. Downsampling is applied to limit the longest dimension of input frames to 768 pixels. Implementation is conducted on PyTorch-1.7.1, and training is executed on a GPU from the NVIDIA GeForce RTX 3060, equipped with 12 GB of video memory. The computational environment utilizes CUDA11.0 for parallel computing.

#### 4.2.3. Inference

DCFA-LVIS is capable of modeling videos of arbitrary lengths without needing to partition the frames into smaller groups. During inference, the entire video is input and then downsampled to 360p, a step that follows the approach of MaskTrack R-CNN [2]. This model learns to represent video-level instances for dynamic segmentation and classification at each individual frame. The decoder is responsible for generating the corresponding box sequences. As a result, there is no requirement for additional post-processing steps related to instances during inference.

### 4.3. Main Results

Table 1 presents a comparison between the DCFA-LVIS model and other leading methods evaluated on the YouTube-VIS 2019 dataset. Among the most advanced approaches, Propose–Reduce [9] utilizes a more powerful backbone network for extracting spatial features and incorporates mask propagation to enhance segmentation and tracking. In contrast, SeqFormer [13] introduces a novel sequence separation decoding strategy and applies Deformable Attention [43] to address both 2D spatial and temporal video features, albeit with the shared drawback of slower inference speeds for both methods.

In Table 1, the outcomes marked with the superscript “Ψ” were derived without simultaneous training on COCO. The top-performing results for models utilizing identical backbone networks are highlighted in bold. The primary objective of this study is to achieve model lightweighting by reducing parameter size and enhancing segmentation speed while maintaining or improving segmentation accuracy. Below is a comprehensive analysis of performance across different backbone networks:

**Performance with ResNet-50 Backbone**
  - **Model with the lowest parameter count**: SipMask has the lowest parameter count (33.2 M), with an FPS of 30.0 and an AP of 33.5%. In contrast, the DCFA-LVIS model proposed in this paper, despite a 31% increase in parameters, achieves a 145% improvement in FPS and a 44% increase in AP, showing a significant enhancement in both speed and accuracy.
  - **Model with the highest FPS**: IFC achieves the highest FPS (107.1) with 39.3 M parameters and an AP of 42.6%. Compared to this, DCFA-LVIS improves AP by 13%, adds 10.9% more parameters, but experiences a 45.7% reduction in segmentation speed.
  - **Model with the highest AP**: DCFA-LVIS achieves the highest AP (48.3%), demonstrating significant advantages in segmentation accuracy.

**Performance with ResNet-101 Backbone**
  - **Optimal model in terms of parameters and accuracy**: DCFA-LVIS achieves 54.5 M parameters and an AP of 49.9%, showing the best parameter efficiency and segmentation accuracy with ResNet-101.
  - **Model with the highest FPS**: IFC achieves the highest FPS (89.4%), with 58.3 M parameters and an AP of 44.4%. In comparison, DCFA-LVIS improves AP by 12.4%, reduces parameters by 6.9%, but experiences a 37% decrease in segmentation speed. Notably, as the backbone depth increases, IFC’s performance advantage diminishes significantly (FPS decreases by 19.7%, parameters increase by 48.3%). In contrast, DCFA-LVIS demonstrates better robustness (FPS decreases by only 13%, parameters increase by 25%, and AP remains high at 49.9%), effectively balancing accuracy and speed when handling large video datasets.

**Performance with DCEResNet Backbone**
  - When using DCEResNet as the backbone, DCFA-LVIS achieves 52.6 M parameters, 65.9 FPS, and an AP of 50.1%. These results validate the comprehensive advantages of the proposed method in terms of accuracy, speed, and parameter efficiency.

**Figure 7:**
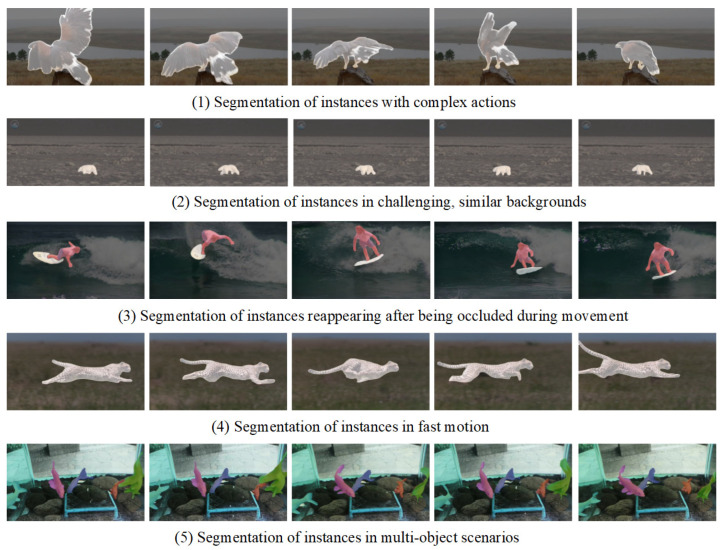
Visualization of DCFA-LVIS on the YouTube-VIS 2019 validation dataset.

Figure 7 provides a visual demonstration of DCFA-LVIS’s performance in four highly challenging scenarios, with detailed comparisons as follows:

- **Instances with complex motions (first row)**: In this scenario, multiple instances exhibit significant motion variations. DCFA-LVIS can precisely capture and track the details of each instance. Despite the complexity of the motions, the masks for each instance remain highly consistent, showcasing their outstanding performance.
- **Indistinguishable similar backgrounds (second row)**: When the background and foreground are highly similar, it is typically difficult to accurately segment the instances. However, DCFA-LVIS successfully segments the foreground instances, avoiding background interference, and demonstrating its exceptional discrimination capability in complex backgrounds.
- **Reappearance of occluded instances (third row)**: After instances are partially or completely occluded, many models may fail to continue tracking them accurately. DCFA-LVIS, however, can accurately resume tracking and segmentation when the instances reappear, highlighting its strong occlusion recovery ability.
- **Instances with rapid movements (fourth row)**: Rapid movements often result in motion blur and deformation, presenting significant challenges for segmentation. DCFA-LVIS maintains precise segmentation even under high-speed motion, proving its efficiency in handling dynamic scenes.
- **Instances in multi-object scenes (fifth row)**: In multi-object scenes where multiple instances exist simultaneously and interfere with each other, the model needs to have strong instance differentiation and tracking capabilities. DCFA-LVIS is able to accurately distinguish between different instances in such complex environments and maintain efficient tracking and segmentation between instances, demonstrating its superior performance in handling multi-object interaction scenarios.

The mask sequences of each instance show a high degree of consistency in the above various complex scenarios, with the same color denoting the same instance, further highlighting the robustness and superiority of DCFA-LVIS in dealing with complex scenarios.

**Figure 8:**
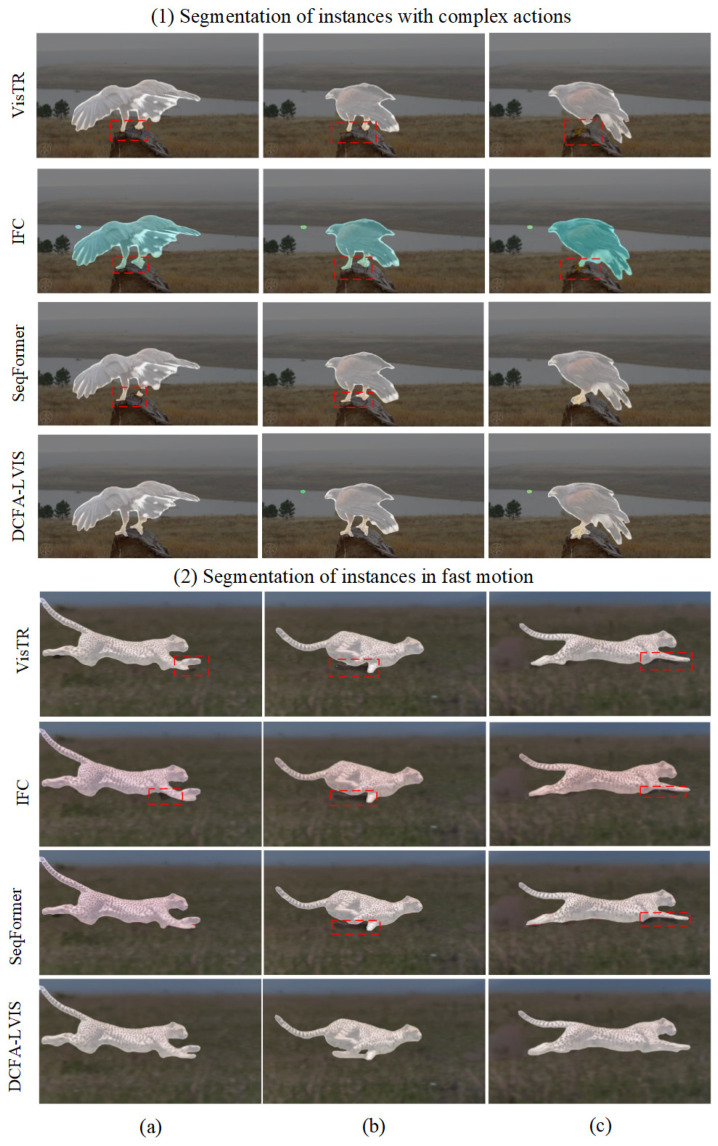
Qualitative comparison with other methods. Each row showcases three frames from a single video, representing different time points, with columns (**a**–**c**) indicating frames captured at the same time instance. The red boxes in the figure highlight areas where the method performs poorly in segmentation results.

Figure 8 provides a visual comparison between the DCFA-LVIS technique and earlier methods like VisTR, IFC, and SeqFormer, all utilizing the ResNet-50 backbone. In this comparison, each row showcases three frames from a single video, representing different time points, with columns (a–c) indicating frames captured at the same time instance.

The comparisons in Figure 8 reveal the following shortcomings of other methods, such as VisTR, IFC, and SeqFormer, in certain scenarios over time:

- **Segmentation detail accuracy**: When handling complex backgrounds or intricate structures, VisTR, IFC, and SeqFormer often exhibit blurred edges or loss of detail. In contrast, DCFA-LVIS demonstrates superior precision in capturing these details, maintaining edge clarity and integrity, particularly excelling in complex scenes.
- **Segmentation performance under occlusion**: For instances that are partially or fully occluded, VisTR, IFC, and SeqFormer frequently produce inaccurate mask predictions upon the instance’s reappearance, leading to misaligned or overlapping instance boundaries. DCFA-LVIS, however, excels at recovering the boundaries of occluded instances, ensuring accurate and consistent masks, highlighting its robust capability to handle occlusion.
- **Instance tracking in high-speed motion scenarios**: In scenes with high-speed motion, VisTR, IFC, and SeqFormer often fail in tracking or produce imprecise segmentations due to motion blur or instance deformation. In comparison, DCFA-LVIS effectively maintains mask continuity and stability when tracking fast-moving instances, significantly reducing segmentation errors and interruptions in tracking.

In summary, the DCFA-LVIS method demonstrates superior segmentation stability and accuracy over time. Whether in handling complex details, recovering from occlusion, or tracking instances in high-speed motion scenarios, it exhibits significant advantages. This robustness enables DCFA-LVIS to achieve outstanding segmentation performance in long video sequences, clearly surpassing other methods.

This study also tests DCFA-LVIS on the newly introduced YouTube-VIS 2021 dataset, which presents greater difficulty due to its increased number of videos, instances, and frames. As shown in Table 2, the model’s top performance with the same backbone network is highlighted in bold. With ResNet-50 as the backbone, DCFA-LVIS achieves an AP of 41.8%, improving by 1.5% compared to the previous method. When using the DCEResNet backbone, the performance reaches 43.9% AP, surpassing the earlier best result with a 2.1% improvement. The proposed methodology in this paper is expected to establish a robust baseline in these benchmarks and contribute to advancing future video instance segmentation research.

### 4.4. Ablation Studies

This section performs a series of ablation tests to examine how various configurations affect the proposed approach. All experiments are carried out using the YouTube-VIS 2019 dataset, instead of models trained on a combination of datasets.

#### 4.4.1. Dual-Channel Feature Enhancement for Adaptive Convolution and Temporal Consistency

Due to the large volume and complexity of video data, instance features often exhibit varying scales and shapes. In such cases, it is essential for the network to adaptively select convolution kernels of different sizes at each pixel position to capture features at various scales. To enhance the flexibility and adaptability of the network, this paper employs a dual-channel feature enhancement mechanism for different input frames. This approach dynamically generates different convolution kernel coefficients and adaptively fuses the kernels, allowing the model to more effectively capture both the internal structure and boundary details of instances, thereby improving instance segmentation accuracy. Furthermore, since this method performs convolution operations only at necessary locations, the idea of adaptively generating weights improves the speed and efficiency of the model compared to standard convolution. Additionally, the dual-channel feature enhancement mechanism helps maintain temporal consistency between video frames. It better adapts to changes in the position and shape of instances across frames, enhancing the continuity and stability of instance segmentation. As shown in Table 3, incorporating the dual-channel feature enhancement mechanism (Dual-Channel-Enhanced, DCE) into the residual network reduces the model parameters by 14.7 M and increases the frames per second (FPS) by 0.7%. While achieving improvements in speed and performance, the model also ensures high-quality accuracy, with the average precision (AP) increasing by 0.9%.

#### 4.4.2. Efficient Instance Query Optimization Based on Dual-Frequency Awareness

The enhancement module that incorporates dual-frequency awareness is crucial to the framework introduced here. Since the same instance may appear at different locations in each frame, it is necessary to independently iteratively refine the spatial sampling regions for each frame. At the same time, this paper also considers that performing attention calculations for each frame in the video would inevitably increase computational overhead, especially in high-resolution videos. Therefore, this paper proposes a time-shared instance query mechanism enhanced by dual-frequency awareness. By utilizing attention mechanisms for both high and low-frequency components, the model captures both detailed local features and broader structural patterns. These features are then merged and passed along to the next processing layers. The grouped attention-capturing idea of the dual-frequency enhancement network enables the learning parameters to be decomposed into two smaller matrices, which helps reduce model parameters and improve efficiency. Meanwhile, the shared instance queries allow for frame-wise query boxes, which remain consistent across every layer of the decoder. These boxes function as stable markers for tracking the same instance throughout the sequence of frames.

In Table 4, the comparison experiments are presented. The findings indicate that the model using the traditional Transformer decoder reaches only 34.1% average precision (AP). This performance is limited by the fact that the same instance query is used across all frames, leading to identical regions being sampled spatially in each frame. This approach fails to accurately represent video-level instances due to its lack of variability and insufficient representation. In contrast, the model with the dual-frequency aware Transformer decoder, which captures local and global features through high-low frequency hierarchical attention, reduces the parameter count by 10.2 M and improves the AP by 13.5%.

#### 4.4.3. Effectiveness of Instance Representation and Mask Head Generalization with Reduced Frame Count

In this experiment, the method aligns and integrates data from each individual frame to create comprehensive instance representations at the video level. To generate these instance representations, this paper attempts to reduce the number of frames used and create a mask head based on these representations. Each frame is processed using this mask head to create a sequence of masks, as illustrated in Table 5. Notably, when only one frame is used as input, this paper achieves an average precision (AP) of 38.6%. When five frames are used as input, the model shows only a 0.5% decrease in average precision (AP) compared to using the entire sequence of frames. This outcome highlights that the mask head generated by our approach performs well on previously unseen frames.

## 5. Conclusions

This study introduces a lightweight video instance segmentation technique, referred to as DCFA-LVIS, designed to address the requirements of visually guided smart sensing. The method enhances the flexibility and accuracy of feature extraction by designing a DCEResNet backbone network that adaptively selects convolution kernels of different sizes at each pixel location to capture features at different scales. Moreover, DCFA-LVIS incorporates a dual-frequency perception enhancement network, enabling the attention mechanism to operate independently on each frame while simultaneously learning a unified query representation for instances at the video level. This innovative application of the idea of capturing attention in groups leads to a more compact model structure and significantly reduces the computational overhead. The evaluation results indicate that DCFA-LVIS outperforms existing methods in both accuracy and speed of video instance segmentation, demonstrating excellent performance and efficiency in complex scenarios. With its lightweight design, the proposed DCFA-LVIS model provides an intelligent, efficient, and practical video processing solution for visual perception intelligent sensing technology. This model significantly enhances the efficiency and adaptability of video data processing, making it suitable for deployment in various practical application scenarios. Optimizing the model’s performance and resource utilization lays a solid foundation for the broader adoption of related technologies across diverse fields.

## Figures and Tables

**Table 1:** Performance metrics for video instance segmentation evaluated on the YouTube-VIS 2019 validation dataset.

Backbone	Method	Params (M)	FPS (%)	AP	AP_50_	AP_75_	AR_1_	AR_10_ (%)
ResNet-50	MaskTrack R-CNN [2]	58.1	20.0	30.1	50.9	32.4	30.8	35.3
	STEm-Seg [8]	50.5	7.0	30.4	50.5	33.3	37.4	36.9
	SipMask [27]	**33.2**	30.0	33.5	53.9	35.6	35.2	39.9
	CompFeat [28]	-	-	35.1	55.8	38.4	32.9	40.1
	VisTR [11]	57.2	69.9	36.0	59.6	36.7	37.0	42.2
	MaskProp [7]	-	-	39.8	-	42.7	-	-
	CrossVIS [29]	37.5	39.8	36.1	56.6	38.7	35.4	40.5
	Propose–Reduce [9]	69.0	-	40.2	62.8	43.6	40.9	49.5
	IFC [12]	39.3	**107.1**	42.6	65.6	46.6	43.6	51.0
	SeqFormer [13]	49.3	72.3	47.4	66.9	51.8	45.5	54.8
	**DCFA-LVIS ^Ψ^**	43.6	73.5	47.6	67.4	52.2	46.2	55.4
	**DCFA-LVIS**	43.6	73.5	**48.3**	**68.2**	**52.8**	**46.4**	**55.9**
ResNet-101	MaskTrack R-CNN [2]	77.2	-	31.6	52.8	33.4	33.0	37.4
	STEm-Seg [8]	69.6	-	34.4	55.6	37.7	34.2	41.4
	VisTR [11]	76.3	57.7	39.9	63.8	44.8	38.1	44.7
	MaskProp [7]	-	-	42.3	-	45.4	-	-
	CrossVIS [29]	56.6	35.6	36.4	57.1	39.5	35.8	41.8
	Propose–Reduce [9]	88.1	-	43.6	65.3	47.2	42.8	53.0
	IFC [12]	58.3	**89.4**	44.4	69.0	49.3	43.8	51.9
	SeqFormer [13]	68.4	64.6	49.0	71.1	55.7	46.8	56.9
	**DCFA-LVIS**	**54.5**	65.0	**49.9**	**72.1**	**56.0**	**47.9**	**58.2**
DCEResNet	**DCFA-LVIS**	**52.6**	**65.9**	**50.1**	**72.7**	**57.0**	**48.1**	**59.4**

**Table 2:** Performance metrics of video instance segmentation on the YouTube-VIS 2021 validation dataset. The top-performing results for models utilizing identical backbone networks are highlighted in bold.

Backbone	Method	Params (M)	FPS (%)	AP	AP_50_	AP_75_	AR_1_	AR_10_ (%)
ResNet-50	MaskTrack R-CNN [2]	58.1	20.0	28.4	48.7	29.4	26.3	33.6
	SipMask [27]	**33.2**	30.0	31.5	52.3	33.8	30.6	37.6
	CrossVIS [28]	37.5	39.8	34.0	54.2	37.7	30.2	38.0
	IFC [12]	39.3	**107.1**	36.4	57.7	39.1	-	-
	SeqFormer [13]	49.3	72.3	40.3	62.2	43.5	35.9	47.9
	**DCFA-LVIS**	43.6	73.5	**41.8**	**63.9**	**44.6**	**37.0**	**49.5**
DCEResNet	**DCFA-LVIS**	**52.6**	**65.9**	**43.9**	**68.4**	**49.3**	**39.2**	**52.0**

**Table 3:** Performance comparison of dual-channel feature enhancement mechanism on DCFA-LVIS model metrics. The bolded parts in the table represent the best results achieved when using the dual-channel feature enhancement mechanism proposed in this paper as the backbone network.

DCE	Params (M)	FPS (%)	AP	AP_50_	AP_75_	AR_1_	AR_10_ (%)
No	68.4	64.6	49.0	71.1	55.7	46.8	56.9
Yes	**53.7**	**65.3**	**49.9**	**71.8**	**56.2**	**47.7**	**58.4**

**Table 4:** Evaluation of performance metrics for the DCFA-LVIS model with dual-frequency-aware transformer decoder. The bolded parts in the table represent the best results achieved when using the dual-frequency-aware mechanism proposed in this paper as the encoder-decoder network.

Variables	Params (M)	FPS (%)	AP	AP_50_	AP_75_	AR_1_	AR_10_ (%)
None	64.7	64.9	34.1	53.7	34.9	34.8	40.9
DFA	**54.5**	**65.0**	**47.6**	**72.1**	**54.7**	**48.0**	**58.0**

**Table 5:** Results of experiments assessing the DCFA-LVIS model’s ability to generalize with varying numbers of input frames.

Frames	AP	AP_50_	AP_75_	AR_1_	AR_10_ (%)
1	38.6	58.8	41.8	39.2	48.0
3	43.9	65.9	48.1	42.9	51.8
5	45.1	67.0	50.2	45.3	55.1
10	45.2	67.4	50.0	44.8	54.0
All	45.6	67.4	51.0	46.1	55.1

## Data Availability

The Youtube-VIS dataset [2,49] is publicly available.

## References

[1] Qi J., Gao Y., Hu Y., Wang X., Liu X., Bai X., Belongie S., Yuille A., Torr P.H.S., Bai S. (2022). Occluded Video Instance Segmentation: A Benchmark. Int. J. Comput. Vis..

[2] Yang L., Fan Y., Xu N. Video instance segmentation. Proceedings of the IEEE/CVF International Conference on Computer Vision.

[3] Zhu X., Wang Y., Dai J., Yuan L., Wei Y. Flow-guided feature aggregation for video object detection. Proceedings of the IEEE International Conference on Computer Vision.

[4] Zhu X., Xiong Y., Dai J., Yuan L., Wei Y. Deep feature flow for video recognition. Proceedings of the IEEE Conference on Computer Vision and Pattern Recognition.

[5] Zhou T., Porikli F., Crandall D.J., Van Gool L., Wang W. (2023). A survey on deep learning techniques for video segmentation. IEEE Trans. Pattern Anal. Mach. Intell..

[6] Voigtlaender P., Krause M., Osep A., Luiten J., Gnana Sekar B.B., Geiger A., Leibe B. MOTS: Multi-object tracking and segmentation. Proceedings of the IEEE/CVF Conference on Computer Vision and Pattern Recognition.

[7] Bertasius G., Torresani L. Classifying, segmenting, and tracking object instances in video with mask propagation. Proceedings of the IEEE/CVF Conference on Computer Vision and Pattern Recognition.

[8] Athar A., Mahadevan S., Osep A., Leal-Taixe L., Leibe B. (2020). STEM-SEG: Spatio-temporal embeddings for instance segmentation in videos. Proceedings of the Computer Vision–ECCV 2020: 16th European Conference.

[9] Lin H., Wu R., Liu S., Lu J., Jia J. Video instance segmentation with a propose-reduce paradigm. Proceedings of the IEEE/CVF International Conference on Computer Vision.

[10] Vaswani A., Shazeer N., Parmar N., Uszkoreit J., Jones L., Gomez A.N., Kaiser L., Polosukhin I. (2017). Attention is all you need. Adv. Neural Inf. Process. Syst..

[11] Wang Y., Xu Z., Wang X., Shen C., Cheng B., Shen H., Xia H. End-to-end video instance segmentation with transformers. Proceedings of the IEEE/CVF Conference on Computer Vision and Pattern Recognition.

[12] Hwang S., Heo M., Oh S.W., Kim S.J. (2021). Video instance segmentation using inter-frame communication transformers. Adv. Neural Inf. Process. Syst..

[13] Wu J., Jiang Y., Bai S., Zhang W., Bai X. (2022). Seqformer: Sequential transformer for video instance segmentation. Proceedings of the European Conference on Computer Vision.

[14] Minaee S., Boykov Y., Porikli F., Plaza A., Kehtarnavaz N., Terzopoulos D. (2020). Image segmentation using deep learning: A survey. IEEE Trans. Pattern Anal. Mach. Intell..

[15] Zhang G., Peng Y., Wang H. (2023). Road traffic sign detection method based on RTS R-CNN instance segmentation network. Sensors.

[16] Wang Q., Song J., Du C., Wang C. (2024). Online scene semantic understanding based on sparsely correlated network for AR. Sensors.

[17] Wu J., Liu Q., Jiang Y., Bai S., Yuille A., Bai X. (2022). In defense of online models for video instance segmentation. Proceedings of the European Conference on Computer Vision.

[18] He K., Gkioxari G., Dollár P., Girshick R. Mask R-CNN. Proceedings of the IEEE International Conference on Computer Vision.

[19] Lin T.Y., Dollár P., Girshick R., He K., Hariharan B., Belongie S. Feature pyramid networks for object detection. Proceedings of the IEEE Conference on Computer Vision and Pattern Recognition.

[20] Chen H., Sun K., Tian Z., Shen C., Huang Y., Yan Y. BlendMask: Top-down meets bottom-up for instance segmentation. Proceedings of the IEEE/CVF Conference on Computer Vision and Pattern Recognition.

[21] Bolya D., Zhou C., Xiao F., Lee Y.J. YOLACT: Real-time instance segmentation. Proceedings of the IEEE/CVF International Conference on Computer Vision.

[22] Huang T., Li H., Zhou G., Li S., Wang Y. (2023). A Survey of Research on Instance Segmentation Methods. J. Front. Comput. Sci. Technol..

[23] Tian Z., Shen C., Chen H., He T. (2019). FCOS: Fully convolutional one-stage object detection. arXiv.

[24] Kirillov A., Wu Y., He K., Girshick R. PointRend: Image segmentation as rendering. Proceedings of the IEEE/CVF Conference on Computer Vision and Pattern Recognition.

[25] Suresha M., Kuppa S., Raghukumar D.S. PointRend segmentation for a densely occluded moving object in a video. Proceedings of the 2021 Fourth International Conference on Computational Intelligence and Communication Technologies (CCICT).

[26] Fang Y., Yang S., Wang X., Li Y., Fang C., Shan Y., Feng B., Liu W. Instances as queries. Proceedings of the IEEE/CVF International Conference on Computer Vision.

[27] Cao J., Anwer R.M., Cholakkal H., Khan F.S., Pang Y. (2020). SipMask: Spatial information preservation for fast image and video instance segmentation. Proceedings of the Computer Vision–ECCV 2020: 16th European Conference.

[28] Fu Y., Yang L., Liu D., Huang T.S., Shi H. CompFeat: Comprehensive feature aggregation for video instance segmentation. Proceedings of the AAAI Conference on Artificial Intelligence.

[29] Yang S., Fang Y., Wang X., Li Y., Fang C., Shan Y., Feng B., Liu W. Crossover learning for fast online video instance segmentation. Proceedings of the IEEE/CVF International Conference on Computer Vision.

[30] Xu K., Yang X., Yin B., Lau R.W.H. Learning to restore low-light images via decomposition-and-enhancement. Proceedings of the IEEE/CVF Conference on Computer Vision and Pattern Recognition.

[31] Chen L., Fu Y., Gu L., Yan C., Harada T., Huang G. (2024). Frequency-aware feature fusion for dense image prediction. IEEE Trans. Pattern Anal. Mach. Intell..

[32] Warren A., Xu K., Lin J., Tam G.K.L., Lau R.W.H. Effective video mirror detection with inconsistent motion cues. Proceedings of the IEEE/CVF Conference on Computer Vision and Pattern Recognition.

[33] Ott M., Edunov S., Grangier D., Auli M. (2018). Scaling neural machine translation. arXiv.

[34] Liu Y., Ott M., Goyal N., Du J., Joshi M., Chen D., Levy O., Lewis Z., Stoyanov V. (2019). RoBERTa: A robustly optimized BERT pretraining approach. arXiv.

[35] Dai Z., Yang Z., Yang Y., Carbonell J., Le Q., Salakhutdinov R. (2019). Transformer-XL: Attentive language models beyond a fixed-length context. arXiv.

[36] Brown T., Mann B., Ryder N., Subbiah M., Kaplan J.D., Dhariwal P., Neelakantan A., Shyam P., Sastry G., Askell A. (2020). Language models are few-shot learners. arXiv.

[37] Sun P., Zhang R., Jiang Y., Kong T., Xu C., Zhan W., Tomizuka M., Li L., Yuan Z., Wang C. Sparse R-CNN: End-to-end object detection with learnable proposals. Proceedings of the IEEE/CVF Conference on Computer Vision and Pattern Recognition.

[38] Meinhardt T., Kirillov A., Leal-Taixe L., Feichtenhofer C. TrackFormer: Multi-object tracking with transformers. Proceedings of the IEEE/CVF Conference on Computer Vision and Pattern Recognition.

[39] Wu J., Jiang Y., Sun P., Yuan Z., Luo P. Language as queries for referring video object segmentation. Proceedings of the IEEE/CVF Conference on Computer Vision and Pattern Recognition.

[40] Arnab A., Dehghani M., Heigold G., Sun C., Lucic M., Schmid C. ViVit: A video vision transformer. Proceedings of the IEEE/CVF International Conference on Computer Vision.

[41] Dosovitskiy A., Beyer L., Kolesnikov A., Weissenborn D., Zhai X., Unterthiner T., Dehghani M., Minderer M., Heigold G., Gelly S. (2020). An image is worth 16x16 words: Transformers for image recognition at scale. arXiv.

[42] Carion N., Massa F., Synnaeve G., Usunier N., Kirillov A., Zagoruyko S. (2020). End-to-end object detection with transformers. Proceedings of the European Conference on Computer Vision.

[43] Zhu X., Su W., Lu L., Li B., Wang X., Dai J. (2020). Deformable DETR: Deformable transformers for end-to-end object detection. arXiv.

[44] Kuhn H.W. (1955). The Hungarian method for the assignment problem. Nav. Res. Logist. Q..

[45] Girshick R. Fast R-CNN. Proceedings of the IEEE International Conference on Computer Vision.

[46] Zheng Z., Wang P., Liu W., Li J., Ye R., Ren D. Distance-IoU loss: Faster and better learning for bounding box regression. Proceedings of the AAAI Conference on Artificial Intelligence.

[47] Milletari F., Navab N., Ahmadi S.A. (2016). V-Net: Fully convolutional neural networks for volumetric medical image segmentation. Proceedings of the 2016 Fourth International Conference on 3D Vision (3DV).

[48] Li B., Yao Y., Tan J., Zhang G., Yu F., Lu J., Luo Y. Equalized focal loss for dense long-tailed object detection. Proceedings of the IEEE/CVF Conference on Computer Vision and Pattern Recognition.

[49] Xu N., Yang L., Yang J., Yue D., Fan Y., Liang Y., Huang T.S. YouTubeVIS Dataset 2021 Version. https://youtube-vos.org/dataset/vis/.

[50] Loshchilov I., Hutter F. (2017). Decoupled weight decay regularization. arXiv.

[51] Lin T.Y., Maire M., Belongie S., Hays J., Perona P., Ramanan D., Dollar P., Zitnick C.L. (2014). Microsoft COCO: Common objects in context. Proceedings of the 13th European Conference on Computer Vision.

